# Cholesterol-Ester Transfer Protein Alters M1 and M2 Macrophage Polarization and Worsens Experimental Elastase-Induced Pulmonary Emphysema

**DOI:** 10.3389/fimmu.2021.684076

**Published:** 2021-07-21

**Authors:** Kelly Gomes Santana, Renato Fraga Righetti, Cristiane Naffah de Souza Breda, Omar Alberto Domínguez-Amorocho, Theresa Ramalho, Francisca Elda B. Dantas, Valéria Sutti Nunes, Iolanda de Fátima Lopes Calvo Tibério, Francisco Garcia Soriano, Niels O. S. Câmara, Eder Carlos Rocha Quintão, Patrícia M. Cazita

**Affiliations:** ^1^ Laboratorio de Lipides, LIM-10, Hospital das Clinicas HCFMUSP, Faculdade de Medicina, Universidade de Sao Paulo, Sao Paulo, Brazil; ^2^ Laboratório de Terapêutica Experimental I (LIM-20), Faculdade de Medicina da Universidade de São Paulo, Sao Paulo, Brazil; ^3^ Transplantation Immunobiology Lab, Department of Immunology, Institute of Biomedical Sciences, Universidade de São Paulo, Cidade Universitária, São Paulo, Brazil; ^4^ Division of Infectious Diseases and Immunology, Department of Medicine, University of Massachusetts Medical School, Worcester, MA, United States; ^5^ Laboratório de Emergências Clínicas (LIM-51), Faculdade de Medicina FMUSP, Universidade de Sao Paulo, Sao Paulo, Brazil

**Keywords:** cholesterol ester transfer protein, macrophage—cell, inflammation, chronic obstructive pulmonary disease, pulmonary emphysema, interleukin-10, arginase 1

## Abstract

Cholesterol-ester transfer protein (CETP) plays a role in atherosclerosis, the inflammatory response to endotoxemia and in experimental and human sepsis. Functional alterations in lipoprotein (LP) metabolism and immune cell populations, including macrophages, occur during sepsis and may be related to comorbidities such as chronic obstructive pulmonary disease (COPD). Macrophages are significantly associated with pulmonary emphysema, and depending on the microenvironment, might exhibit an M1 or M2 phenotype. Macrophages derived from the peritoneum and bone marrow reveal CETP that contributes to its plasma concentration. Here, we evaluated the role of CETP in macrophage polarization and elastase-induced pulmonary emphysema (ELA) in human CETP-expressing transgenic (huCETP) (line 5203, C57BL6/J background) male mice and compared it to their wild type littermates. We showed that bone marrow-derived macrophages from huCETP mice reduce polarization toward the M1 phenotype, but with increased IL-10. Compared to WT, huCETP mice exposed to elastase showed worsened lung function with an increased mean linear intercept (Lm), reflecting airspace enlargement resulting from parenchymal destruction with increased expression of arginase-1 and IL-10, which are M2 markers. The cytokine profile revealed increased IL-6 in plasma and TNF, and IL-10 in bronchoalveolar lavage (BAL), corroborating with the lung immunohistochemistry in the huCETP-ELA group compared to WT-ELA. Elastase treatment in the huCETP group increased VLDL-C and reduced HDL-C. Elastase-induced pulmonary emphysema in huCETP mice promotes lung M2-like phenotype with a deleterious effect in experimental COPD, corroborating the *in vitro* result in which CETP promoted M2 macrophage polarization. Our results suggest that CETP is associated with inflammatory response and influences the role of macrophages in COPD.

## Introduction

CETP is an independent risk factor for the development of atherosclerosis. However, inhibition of CETP as a therapeutic strategy for raised HDL-C and as a protection against cardiovascular disease failed to show benefits in population-based investigations. In this regard, surprisingly, a CETP inhibitor (Torcetrapib) in the ILLUMINATE trial increased the frequency of infection cases, although this did not occur with other inhibitors (1). This undesirable effect is not attributable to a specific CETP inhibition but to secondary changes in plasma lipoprotein metabolism or an elevation of plasma aldosterone (2).

In animal models, CETP inhibition protected transgenic mice expressing human CETP (1) as well as rabbits naturally expressing CETP against atherosclerosis (3, 4). The CETP link with atherosclerosis and infection has been reviewed in humans (5), and lower plasma CETP concentrations occurred in patients who did not survive sepsis compared to survivors. Additionally, a positive correlation between plasma CETP concentration and sepsis survival rate was reported (6). CETP is involved in the inflammatory response in mice expressing the human CETP gene (huCETP), which is more resistant to endotoxemia and experimental sepsis than in wild type mice (5, 7, 8). However, these results diverge from more recent results concluding that human CETP worsens inflammation and sepsis in mice (9), with CETP inhibition improving the survival rate in human sepsis (10, 11). Although there is no explanation for these divergent results, it is possible to conjecture that the role of CETP could also depend on the animal species and pathology involved since CETP inhibition protects against hepatic complications in *Schistosomiasis japonicum* (12). However, it could also have tissue-specific roles.

Although several risk factors are present in sepsis, functional alterations in LP metabolism and immune cell populations, including macrophages, may worsen the disease. CETP synthesis occurs primarily in macrophage-rich tissues, such as the spleen and liver, and is secreted into the blood, with a greater contribution from Kupffer cells in humans and huCETP mice (13). The latter study showed co-localization between CETP and macrophage surface markers such as CD68. In mice, both peritoneal and bone marrow-derived macrophages were associated with the CETP expression, with bone marrow-derived CETP being a significant contributor to the activity and total concentration of CETP in plasma (14).

An important question remains about the function of CETP production by macrophages where activated macrophages are common features of many inflammatory diseases. CETP belongs to the family of lipid transfer/lipopolysaccharide-binding proteins (15). Moreover, administration of lipopolysaccharide to hamsters, animals that express CETP, or transgenic mice expressing human CETP, induces a rapid decrease in serum CETP. This suggests that CETP, which has molecular similarity to lipopolysaccharide-binding proteins, could modulate the lipopolysaccharide response and play a role in innate immunity and cholesterol metabolism (14).

In the present investigation, the macrophage phenotypic profile was evaluated in the presence and absence of human CETP (huCETP). HuCETP expression in mice inhibits *in vitro* macrophage polarization to the M1 phenotype and promotes M2 phenotype. We further demonstrated in an experimental model of pulmonary emphysema that huCETP mice exposed to elastase have M2 phenotype macrophages in the lung tissue and that its functional parameter worsens. Our results raise the possibility of CETP participating in the inflammatory response and the macrophage roles in COPD.

## Material and Methods

### Animal Model

The experimental protocol followed the Ethical Principles in Animal Experimentation of the Brazilian Society for Laboratory Animal Science (SBCAL) and was approved by the University of São Paulo Medical School Ethics Committee the use of animals (Comissão de Ética no Uso de Animais: CEUA 949/2018) according to the ARRIVE research guideline for the use of Laboratory animals (16). Human CETP transgenic (Tg) mice (line 5203, C57BL6/J background) expressing human CETP under the control of its own promoter and other major regulatory elements were developed by Dr. Alan Tall (Department of Medicine, Columbia University, New York, NY, USA). A human CETP minigene with 3.4-kb (5′) and 2.2-kb (3′) natural flanking sequences was inserted (17) and kindly provided by Professor Helena C.F. Oliveira (University of Campinas, São Paulo, Brazil). Hemizygous human CETP Tg mice were cross bred to generate huCETP mice, and non-transgenic wild type (WT) mice were used as controls. The presence of the targeted alleles, the CETP transgene, was determined according to the Jackson Laboratory modified protocol for the B6.CBA-Tg (CETP) 5203Tall/J line, number 3904 [https://www.jax.org/Protocol?stockNumber=003904 (see **Supplementary (S) Materials Figure S1**]. The CETP activity and lipoprotein profile were also evaluated (**Supplementary Figures S2**, **S3**). Male CETP and WT mice, between 8 and 12 weeks of age, were housed in a conventional animal facility in a temperature-controlled room (22 ± 1°C), on a 12-h light/12-h dark cycle, with free access to water and food, and placed on a standard rodent chow diet (Quimtia CR1, Colombo, PR, Brazil). The plasma CETP levels measured by ELISA (µg/ml ± SD) were 5.80 ± 2.07 and 0.02 ± 0.02 for CETP Tg mice and WT mice, respectively (8).

### Isolation of Bone-Marrow-Derived Macrophages

Bone marrow cells were harvested from WT or CETP Tg mice after anesthesia and exsanguination as previously described (18). Briefly, under aseptic conditions and after dissection of the hind limb long bones (femurs and tibiae), the knee joint was removed with scissors at the proximal end. Then, a 10 ml syringe with 26G needle containing Dulbecco’s Modified Eagle Medium (DMEM), high glucose, 10% fetal bovine serum (FBS) (Invitrogen), and 1% penicillin/streptomycin] was inserted to wash the bone marrow and obtain the gelatinous tissue that fills the medullary cavities, which was then placed in 50 ml Falcon^®^ centrifuge tubes. Cell aggregates were centrifuged (6 min, 1,000 rpm) at room temperature; thereafter, cells were seeded in six-well culture plates and maintained for 7 days at 37°C in a humidified atmosphere containing 5% CO_2_. On day 5, the DMEM was supplemented with 30% of growth supernatant of M-CSF-transduced L929 (18).

### Macrophage Polarization in the Presence or Absence of Human CETP

Heterogeneity is one of the most important macrophage characteristics. To investigate the CETP effect on macrophage (M) polarization, we used huCETP Tg bone marrow cells and compared then to macrophages that do not produce CETP from non-transgenic littermate (WT) mice.

Cells were cultured in high glucose DMEM supplemented with 10% FBS, 1% penicillin/streptomycin under stimulus conditions with 5 ng/ml IFN-*γ* (PeproTech, USA), plus 50 ng/ml LPS (055:B5, Sigma-Aldrich) for induction of M1, or 10 ng/ml IL-4 (PeproTech, USA), plus 10 ng/ml IL-13 (PeproTech, USA) for M2 polarization at 37°C in a humidified atmosphere containing 5% CO_2_ for 24 h. M0 macrophages were maintained in DMEM without polarization factors.

A second experiment was performed in which exogenous CETP (Recombinant human CETP, Roar Biomedical, New York, NY, USA) was added to the culture medium (1 µg/ml) in the bone marrow-derived macrophages from WT donors. The culture was performed in the same conditions as described above.

### Gene-Expression Analysis

After treatments, bone marrow-derived macrophages from WT or CETP mice were washed with phosphate buffered saline (PBS), removed with a scraper from the culture plate, and centrifuged at 1,000 rpm (4°C) to obtain the cell pellet. Total RNA was isolated from cell lysates following the manufacturer’s instructions (RNeasy^®^ Mini Kit; Qiagen, Hilden, Germany) and retrotranscribed with the High Capacity cDNA reverse transcription kit (Applied Biosystems, Foster City, CA, USA). Quantitative PCR was performed using the StepOne Plus™ Real Time PCR system (Applied Biosystems) with TaqMan Universal Master Mix II (Thermo Fisher, Waltham, MA, USA) and SYBR probes plus the Master Mix Power SYBR™ Green solution (Applied Biosystems). The method chosen to calculate the relative quantification was the comparative Ct (cycle threshold) (ΔΔCt). FAM-labeled TaqMan probe detection and the primer assay designed for SYBR^®^ Green (Applied Biosystems, Foster City, CA, USA) were used for gene expression quantitative real-time PCR analysis. *β*-actin was the endogenous control. The codes and sequences are listed in **Supplementary Table S1**.

### Flow Cytometry Analysis

After 24 h, cells were washed with ice-cold PBS and removed from the surface of the plate with 1 ml of accutase solution (Sigma-Aldrich), placed gently on the plate, and maintained for 5 min. The cell suspension was transferred to a 15 ml sterile conical tube and centrifuged at 300 × g (4 min) to obtain the cell pellet. The supernatant was completely removed by rapid decanting, and the cell pellet was immediately resuspended in 200 µl of PBS containing 2% FBS. The live cells were analyzed using the Live/Dead marker (ThermoFischer L34966). Cells were incubated with fluorescence-conjugated antibodies against the surface markers CD11b (APCCy7)/F4/80 (PercP) (BioLegend, San Diego, CA), in PBS containing 1% BSA for 30 min at 4°C. CD80 (APC) was used as a marker for M1, and CD206 (PE) was used for M2 macrophages (BioLegend, San Diego, CA). CD80^+^/F4/80^+^/CD11Bc^+^/CD206^−^ cells were marked as M1-positive cells, while CD80^−^/F4/80^+^/CD11Bc^+^/CD206**^+^** cells were marked as M2-positive cells. All samples were analyzed using a flow cytometer (BD FACSDiva™ software; Biosciences, San Jose, CA). A total of 100,000 events were collected for each sample, and the data were analyzed using FLOWJO (v. 8.7) software (Tree Star Inc., Ashland, OR, USA) (19, 20).

### Cytokines and CETP Protein Analysis

The protein levels of TNF, IL-6, IL-10, and CETP (plasma, cell supernatants, and bronchoalveolar lavage: BALF) were measured using a commercially available enzyme-linked immunosorbent assay (ELISA) kit (R&D Systems, Minneapolis, MN) and by Cell Biolabs (Cell Biolabs, Inc., San Diego, CA, USA), according to the manufacturer’s instructions.

### Induction of Emphysema: Elastase-Induced Model

WT and CETP animals were anesthetized with isofluorane, and the trachea was exposed after asepsis of the anterior neck region. Porcine pancreatic elastase (PPE) (Type 1/E1250; Sigma-Aldrich, El Camino Real Carlsbad, CA 92009, California, USA) at a single dose of 0.667 IU (50 µl saline solution) was administered intratracheally between the cartilaginous rings. The control group (no elastase) received only 50 µl of saline solution.

### Respiratory Mechanics Analysis

After 28 days, animals were anesthetized, tracheostomized, and placed on a rodent mechanical ventilator (flexiVent, SCIREQ, Montreal, Canada) with a tidal volume of 10 ml/kg and a respiratory rate of 120 cycles/min. Respiratory mechanics were performed, comprising respiratory system resistance (Rrs), tissue elastance (Htis), lung tissue resistance (Gtis), respiratory system elastance (Ers), and airway resistance (RAW), using the previously described model by (21). The exhaled nitric oxide (ENO) concentration was measured by collection Mylar bags attached to the expiratory output of the ventilator for 10 min, and an NO filter was attached to the inspiratory breathing circuit input. Afterwards, a chemiluminescence fast-responding analyzer (NOA 280; Sievers Instruments Inc., Boulder, CO, USA) calibrated with an NO (nitric oxide), source certified 47-parts per billion (ppb) (White Martins, São Paulo, Brazil) and a zero NO filter (Sievers Instruments Inc.) was used.

### Bronchoalveolar Lavage

The lungs were washed with 0.9% saline (3× 0.5 ml). Volume recovery of the instilled saline was over 95%, which was transferred into a test tube on ice. White blood cells were quantified through total and differential counting. BAL was centrifuged at 1,000 rpm for 10 min, and the cell pellet was rediluted in 0.2 ml sterile saline. The total number of viable cells was determined in a Neubauer hemocytometer counting chamber (400×). The differential cell counts were performed in cytocentrifuge preparations of the BAL (450 rpm for 6 min) (Cytospin, Cheshire, UK) stained with Diff-Quick (Biochemical Sciences Inc., Swedesboro, NJ). Three hundred cells (macrophages, lymphocytes, eosinophils, and neutrophils) were counted in each Dif Quick-stained cytospin slide in a blinded fashion.

### Lung Histology

After the respiratory mechanic assessment, mice were euthanized by abdominal aorta exsanguination; lungs were removed and fixed at a constant pressure (20 cm H_2_O) using 10% buffered formalin infused through the trachea for 48 h. For structure analysis, the lungs were embedded in paraffin, cut into 5-μm coronal sections for morphometry evaluation, and stained with H&E.

### Immunohistochemistry

Tissue sections were deparaffinized and rehydrated. Immunohistochemistry analysis was performed as previously described (21). Endogenous peroxidases were blocked by incubation with 3% hydrogen peroxide (H2O2) 10 V (3 × 10 min), and slide sections of experimental groups were incubated overnight at 4°C with primary antibodies. Afterwards, they were washed with phosphate buffered saline (PBS), incubated with secondary antibodies at 37°C, and stained with chromogenic solution (DAB). The Harris’ hematoxylin cell number positive for TNF, IL-10, iNOS, and CETP in the lung parenchyma was measured by counting number of spots. Dilution of the antibodies was as suggested by the manufacturer and described in **Supplementary Table S2**.

### Mean Linear Intercept

The alveolar diameter analysis was performed using a reticule of known area (50 lines and 100 points) coupled in a microscope (Olympus CH30) ×400. Slides were stained with hematoxylin and eosin (H&E) for morphometric determination. The reticule was placed over the area of the lung parenchyma, and the intersections of the points of the alveolar wall were counted, excluding vessels and airways. Thus, the average alveolar diameter was calculated, reasoning that the number in contact with the alveolar walls is also the number of intersections between lines and alveolar walls (22). The mean linear intercept (LM) was quantified by one observer in a blinded fashion to assess air space enlargement (21).

### Statistical Analysis

Continuous variables were tested for normality with the Kolmogorov–Smirnov and Shapiro Wilk tests. The values are expressed as median and percentiles 25 ant 75 or as the mean and standard deviation for non-parametric or parametric data respectively. Non-parametric data were compared using the Mann–Whitney U test for two independent samples or Kruskal–Wallis test with original FDR method of Benjamini and Hochberg post-test for three or more samples. Parametric data were compared by Student’s t-test for two independent samples or ANOVA with two-stage linear step-up procedure of Benjamini, Krieger, and Yekutieli post-test for three or more samples (23–25). Graphs were prepared using GRAPHPAD PRISM version 9.0 (GraphPad Software, San Diego, CA, USA). Statistical significance was set at p <0.05.

## Results

### *In Vitro* Studies

#### Polarization of Macrophages in the Presence and Absence of CETP

**Figure 1:**
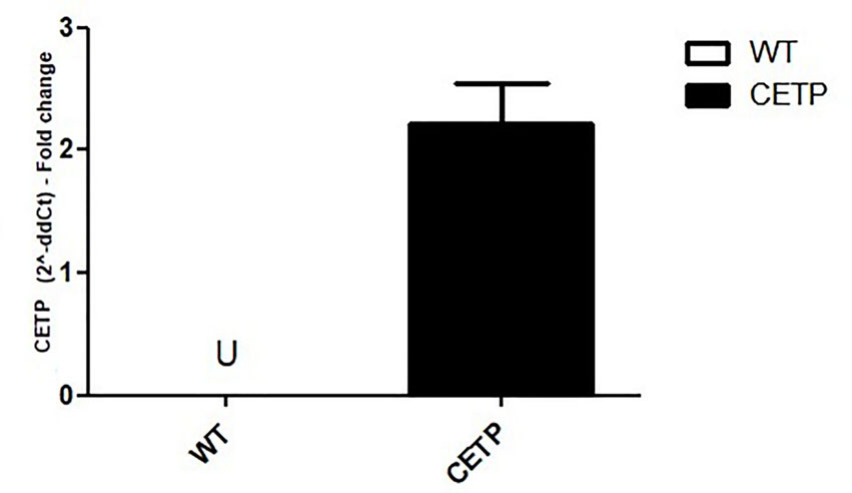
Comparison of CETP mRNA expression in bone marrow-derived macrophages from WT and huCETP Tg mice. mRNA expression was determined using RT-PCR. Results are expressed as the mean ± standard deviation. U, undetermined.

To investigate the effect of CETP on the M1/M2 macrophage polarization, we confirmed that bone marrow (BM)-derived macrophages from CETP transgenic (CETP-Tg) express the human CETP transgene compared to their non-transgenic wild type (WT) littermate mice. CETP mRNA expression was not detected in WT cells (**Figure 1**).

**Figure 2:**
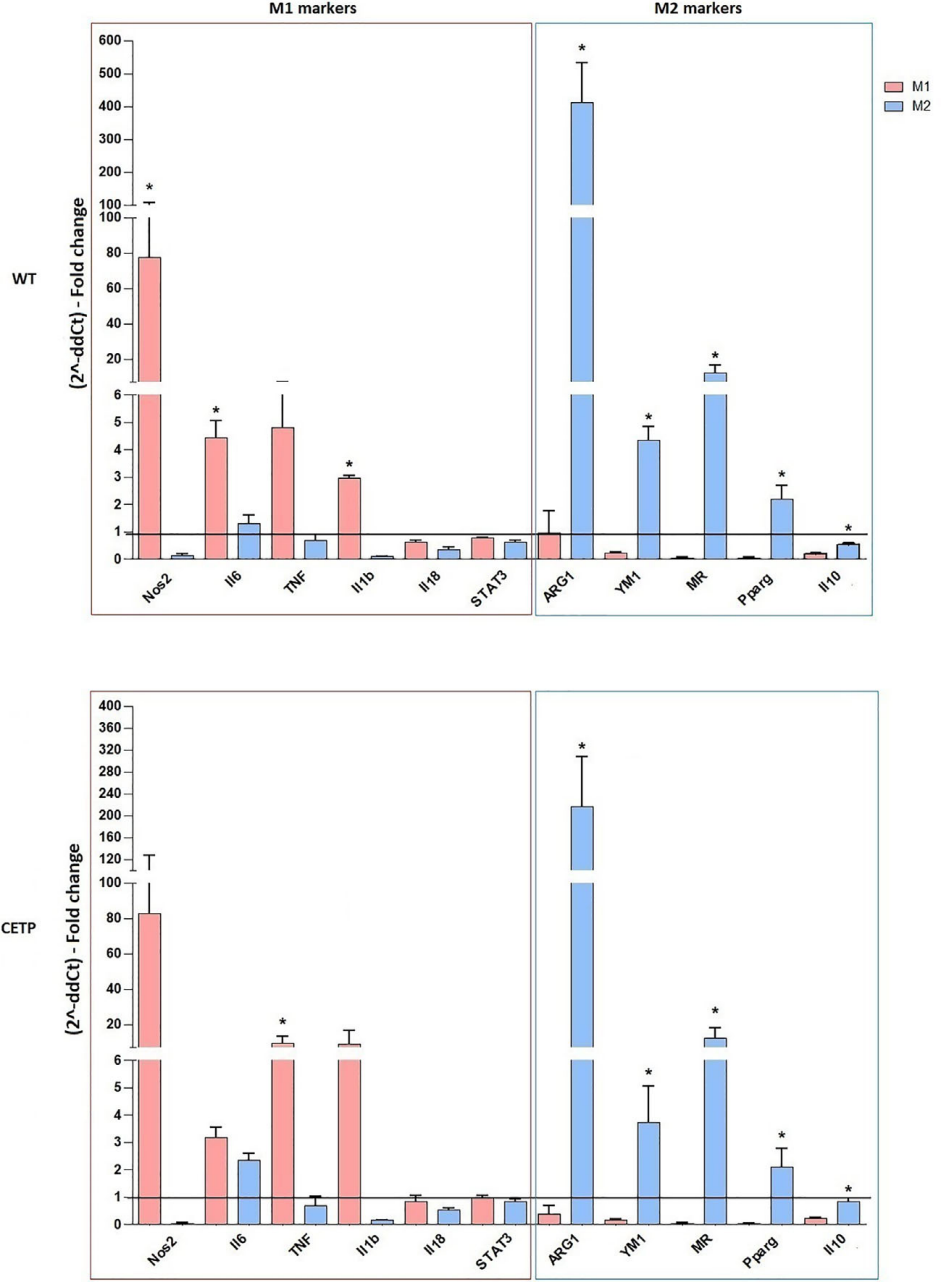
Gene expression of typical M1 and M2 markers from bone marrow-derived macrophages of WT and huCETP Tg mice. *In vitro* stimulated M1 (5 ng/ml IFN*γ* + 50 ng/ml LPS) and M2 (10 ng/ml IL-13 + IL-4) for 24 h. mRNA expression was determined using RT-PCR. Results are expressed as the mean ± standard deviation and were compared by unpaired Student’s t-test (n = 3–5). *P < 0.05.

Macrophages can also be generated from monocytes in vitro and undergo classical M1 (LPS + IFN-γ) or alternative M2 (IL-4 + IL-13) activation. Previous studies have reported differences in gene expression between M1 and M2 macrophages grown in vitro compared to non-activated macrophages (M0). M1 responds by positively regulating many pro-inflammatory genes according to the LPS stimulus, while induction to M2 promotes anti-inflammatory genes such as arginase-1 (26). Here, we examined the primary genes representing the degree of polarization for M1 or M2 in macrophage CETP production (CETP endogenous) compared to the WT (**Figure 2**). Stimulation for M1, as expected, increased NOS2, TNF, IL-6, and IL-1β gene expression (*P* < 0.05; M1 *vs.* M2). Expressions of arginase (ARG1), YM1, mannose receptor CD206 (MR), peroxisome proliferator-activated receptor (PPAR) gamma, and IL-10, considered markers of M2, were higher in this population although not differing between WT and CETP groups (**Figure 2**).

**Figure 3:**
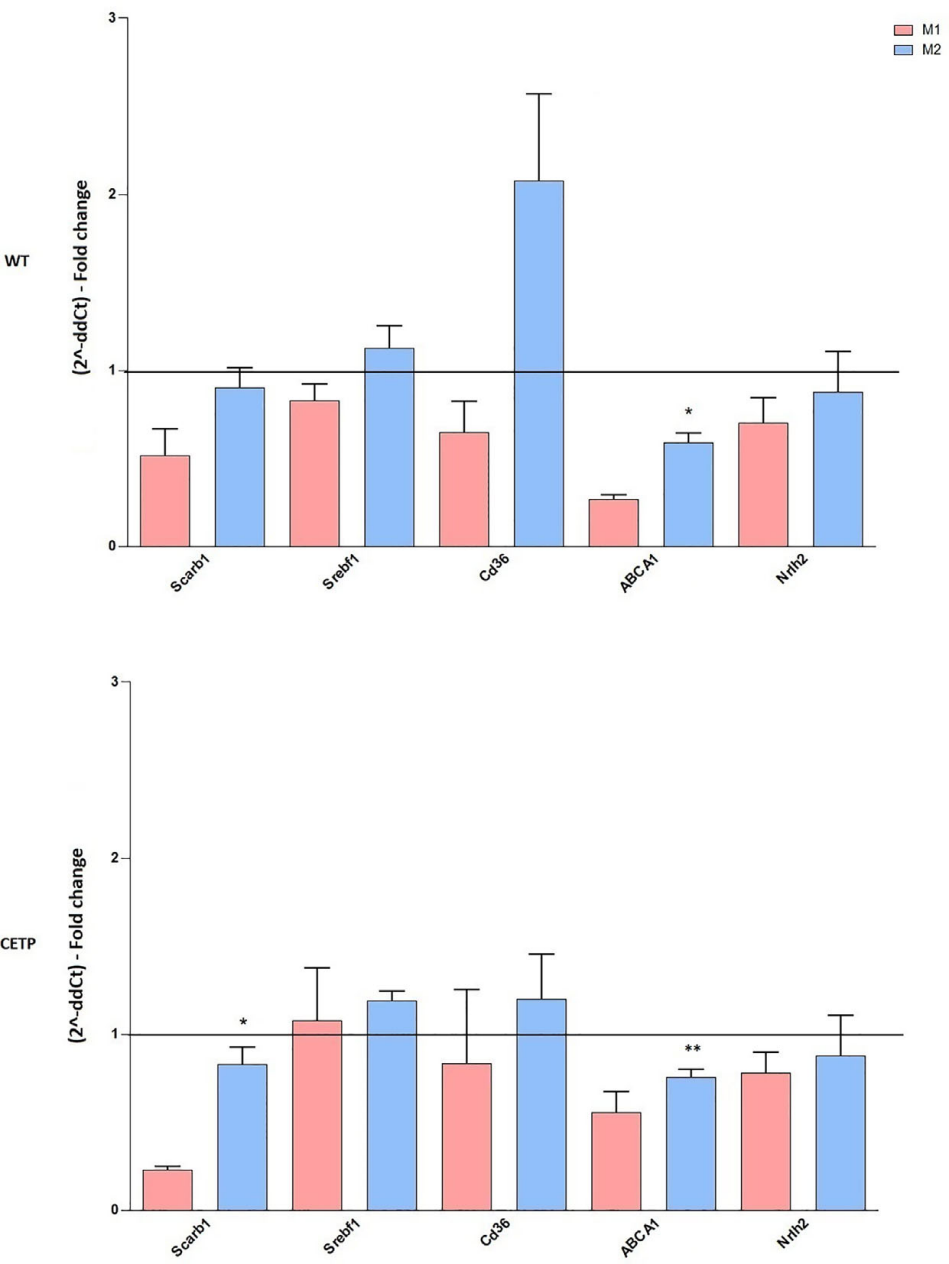
Gene expression of factors associated with lipid metabolism from bone marrow-derived macrophages of WT and huCETP Tg mice. *In vitro* stimulated M1 (5 ng/ml IFN*γ* + 50 ng/ml LPS) and M2 (10 ng/ml IL-13 + IL-4) for 24 h. mRNA expression was determined using by RT-PCR. Results are expressed as mean ± standard deviation and were compared by unpaired Student’s t-test. (n = 3–5). *P < 0.05 (M1 *vs*. M2); **P < 0.05 (CETP M2 *vs*. WT M2).

Considering that CETP plays an essential role in the metabolism of lipids and lipoproteins due to inflammation and lipid signaling interconnections, we analyzed mRNA expressions of lipid metabolism genes. Increased ABCA1 expression was observed in M2 compared to M1 in both CETP and WT cells. Furthermore, CETP-M2 cells expressed slightly increased ABCA1 than WT-M2 did and increased expression of SRB1 (SCARB1) compared to CETP-M1 (**Figure 3**).

**Figure 4:**
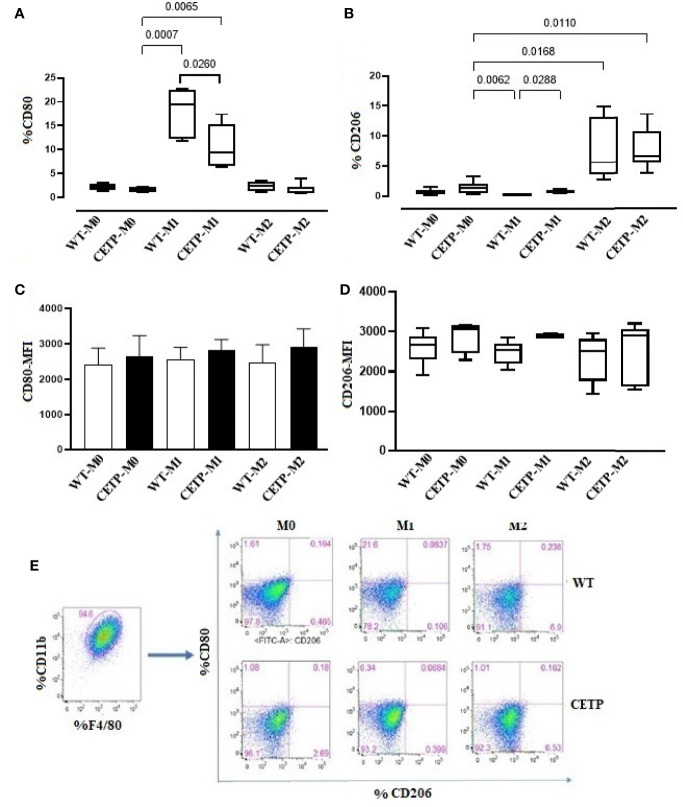
Expression of cell surface markers associated with M1 (CD80) and M2 (CD206) from bone marrow-derived macrophages of WT and huCETP Tg mice. Expression of CD80 **(A)**, CD206 **(B)**, mean fluorescence intensity (MFI) of CD80 **(C)**, MFI of CD206 **(D)**, and representation of the gate strategy **(E)**. Non-stimulated control cells (M0), stimulated M1 (5 ng/ml of IFN*γ* + 50 ng/ml of LPS) or M2 (10 ng/ml of IL-13 + IL-4) for 24 h. Basal fluorescence was determined using unlabeled cells, and compensation was performed with cells labeled with the respective fluorochromes on the FACSCanto II cytometer. In total, 100,000 events were analyzed. Values were expressed as mean and standard deviation. Values expressed as median and percentiles 25 and 75 were analyzed by Kruskal–Wallis test with original FDR method of Benjamini and Hochberg post-test. The values expressed as mean and standard deviation were analyzed by ANOVA with two-stage linear step-up procedure of Benjamini, Krieger, and Yekutieli post-test; (n = 3), P < 0.05.

We also evaluated cell surface markers associated with M1 (CD80) and M2 (CD206). The percentage of expression of CD80 was higher in the WT-M1 than in CETP-M1 macrophages (**Figure 4A**). CETP macrophages increased the expression of CD206 in cells in the absence of stimulus (M0) and under stimulation for M1 (IFN*γ* + LPS) in comparison to WT-M1 (**Figure 4B**). The mean fluorescent intensity (MFI) in flow cytometry analysis did not differ between groups (**Figures 4C–E**).

**Figure 5:**
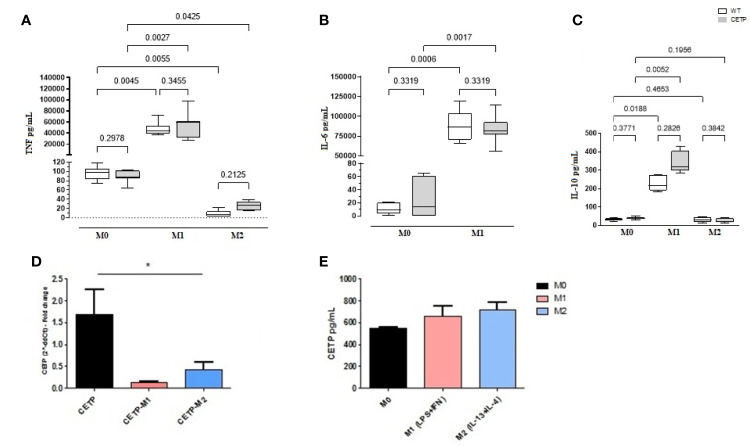
Cytokine secretion, CETP expression, and secretion in cell culture medium. Bone marrow-derived macrophages from WT and huCETP mice, unpolarized (M0), or stimulated to M1 (5 ng/ml IFN*γ* + 50 ng/ml LPS) or to M2 (10 ng/ml IL-13 + IL-4) for 24 h. TNF-α **(A)**, IL-6 **(B)**, IL-10 **(C)**, CETP mRNA expression in macrophages **(D)** and CETP secretion **(E)**. Concentrations were determined by ELISA, and CETP expression was determined by RT-PCR. Values expressed as median and percentiles 25 and 75 were analyzed by Kruskal–Wallis test with original FDR method of Benjamini and Hochberg post-test. Values were expressed as mean and standard deviation analyzed by ANOVA with two-stage linear step-up procedure of Benjamini, Krieger, and Yekutieli post-test; (n = 5–8). P < 0.05. *M0 *vs*. M1 and M2.

IFN*γ* and toll-like receptor (TLR) agonists, such as LPS, are the primary stimuli generating M1 macrophages that express inflammatory cytokines, such as TNF, whereas IL-4 and IL-13 are mainly used to generate M2 macrophages resulting in high production of IL-10. Analysis of the cell culture supernatants showed higher TNF, IL-6, and IL-10 cytokines in M1 (CETP and WT), confirming the inflammatory stimulus (**Figures 5A–C**). Secretion of CETP in the culture medium was similar in all treatment conditions (**Figure 5E**). However, CETP expression was downregulated by the inflammatory stimulus (**Figure 5D**).

**Figure 6:**
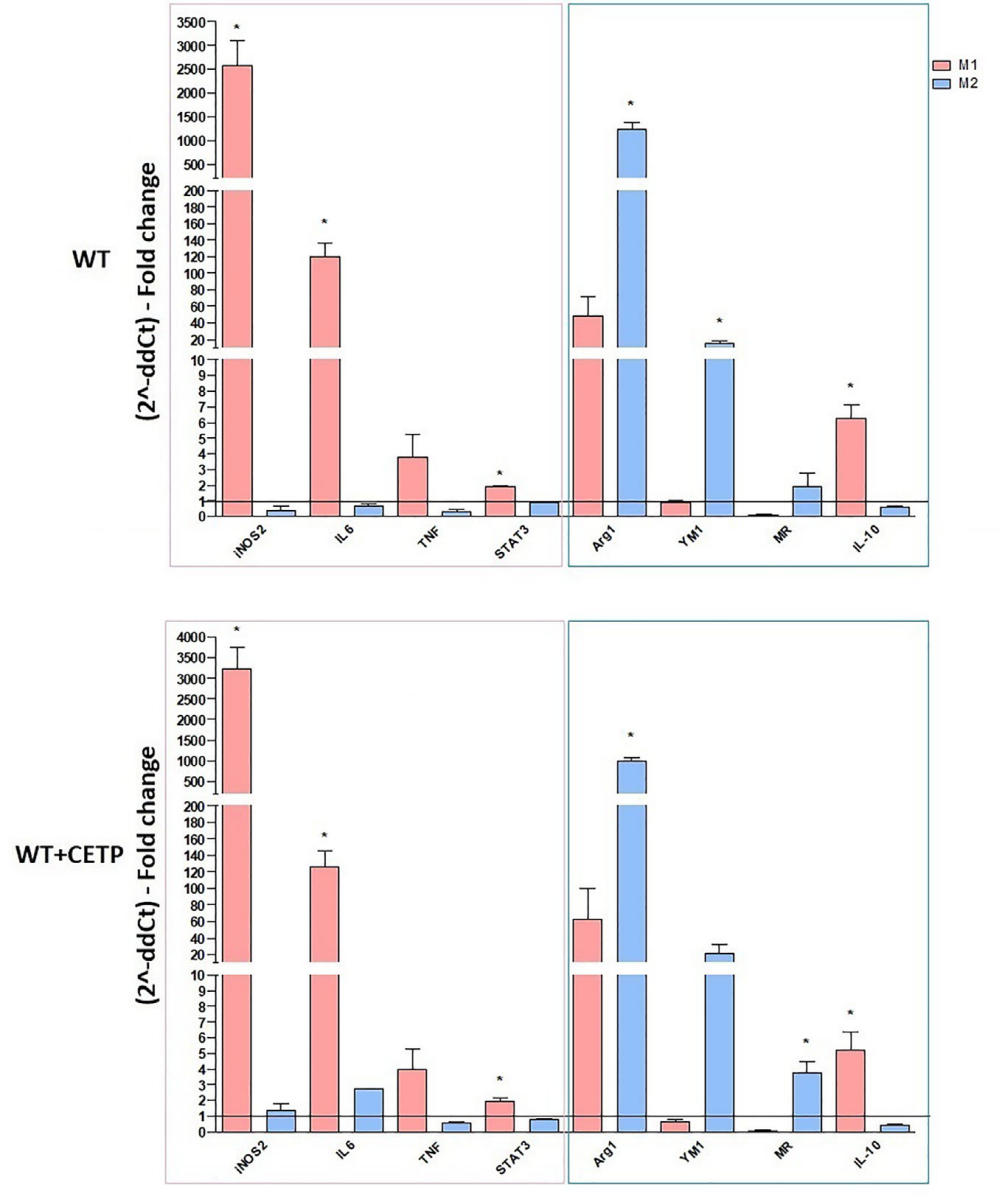
Gene expression of typical M1 and M2 markers in bone marrow-derived macrophages from WT mice stimulated in the presence of recombinant CETP *in vitro*. Macrophages were stimulated using 5 ng/ml IFN*γ* + 50 ng/ml LPS (M1) or 10 ng/ml IL-13 + IL-4 (M2) for 24 h. mRNA expression was determined by RT-PCR. Results are expressed as the mean ± standard deviation and compared by unpaired Student’s t-test. (n = 3–5). *P < 0.05 for 24 h.

**Figure 7:**
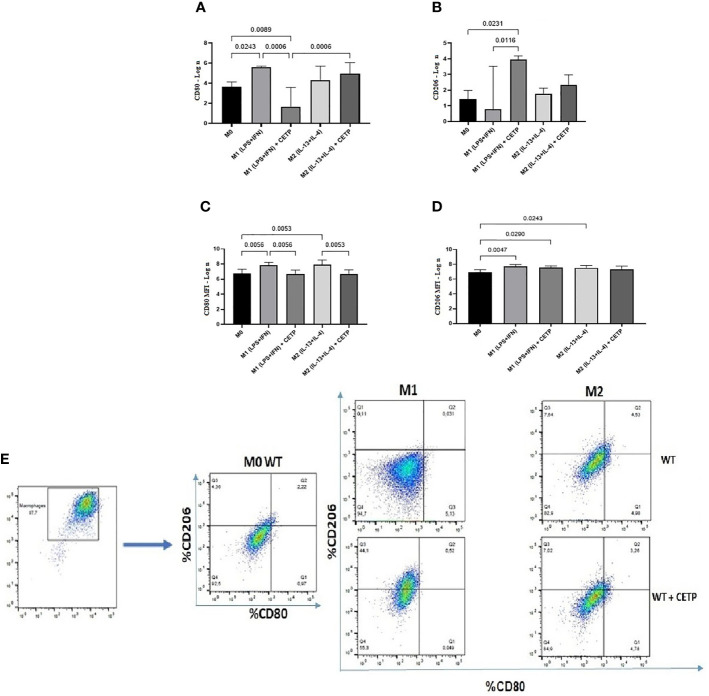
Expression of cell surface markers associated with M1 (CD80) and M2 (CD206) from bone marrow-derived macrophages of WT mice in the presence of recombinant CETP. Expression of CD80 **(A)**, CD206 **(B)**, mean fluorescence intensity (MFI) of CD80 **(C)**, MFI of CD206 **(D)**, and representation of the gate strategy **(E)**. Non-stimulated control cells (M0), stimulated M1 (5 ng/ml of IFN*γ* + 50 ng/ml of LPS) or M2 (10 ng/ml of IL-13 + IL-4) for 24 h. Basal fluorescence was determined using unlabeled cells, and compensation was performed with cells labeled with the respective fluorochromes on the FACSCanto II cytometer. In total, 100,000 events were analyzed. Values were expressed as mean and standard deviation. These data were analyzed by ANOVA with two-stage linear step-up procedure of Benjamini, Krieger, and Yekutieli post-test; (n = 3). P < 0.05.

**Figure 8:**
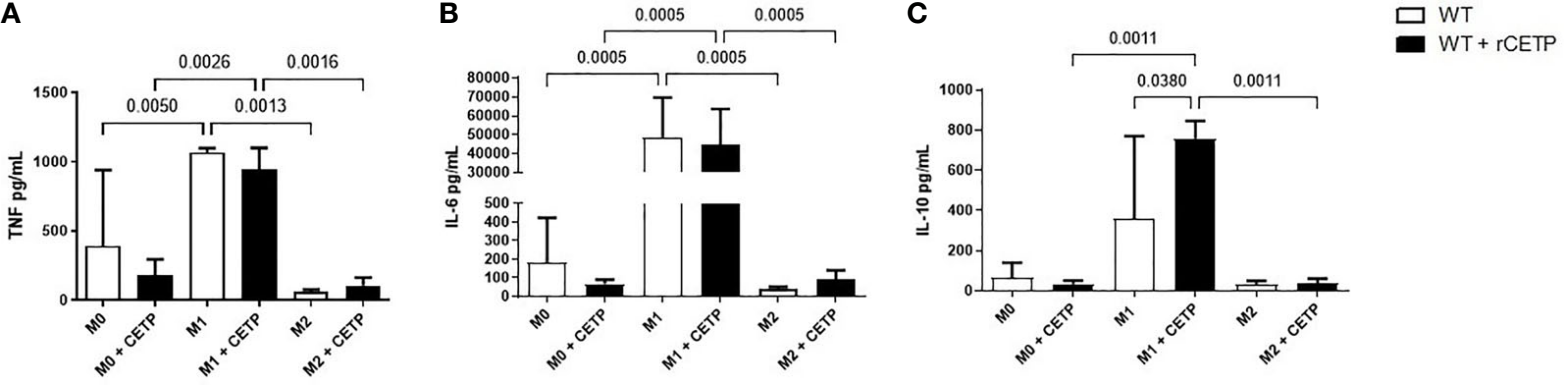
Cytokine secretion in cell culture medium. Bone marrow-derived macrophages from WT mice, unpolarized (M0), or stimulated to M1 (with 5 ng/ml IFNg + 50 ng/ml LPS), or to M2 (with 10 ng/ml IL-13 + IL-4) in the absence (control) or presence of recombinant human CETP (1 µg/ml) for 24 h. TNF **(A)**, IL-6 **(B)** and IL-10 **(C)**.

Next, we investigated the influence of exogenous CETP (human recombinant CETP; rCETP) added to the culture medium. Generally, gene expressions of a few M1 or M2 markers and cytokines in supernatants collected from cell culture were similar to those found in the macrophages that produce CETP (endogenous) (**Figures 6** and **8**). However, we observed increased MR (CD206) gene expressions in the presence of rCETP under stimulation for M2 (**Figure 6**). Reduction in the CD80 (**Figures 7A, C**) M1 marker and increased CD206 percent expression (**Figure 7B**) occurred in the presence of rCETP compared to WT. Increased CD206 MFI was observed in comparison to M0 (**Figure 7D**). **Figure 7E** shows the gate strategy. Cell culture supernatants showed higher TNF, IL-6, and IL-10 cytokines in M1 (CETP and WT) (**Figures 8A–C**). However, IL-10 production was higher in CETP-M1 in comparison to WT-M1 (**Figure 8C**).

### *In Vivo* Studies

#### Induction of Emphysema: Elastase-Induced Model

**Figure 9:**
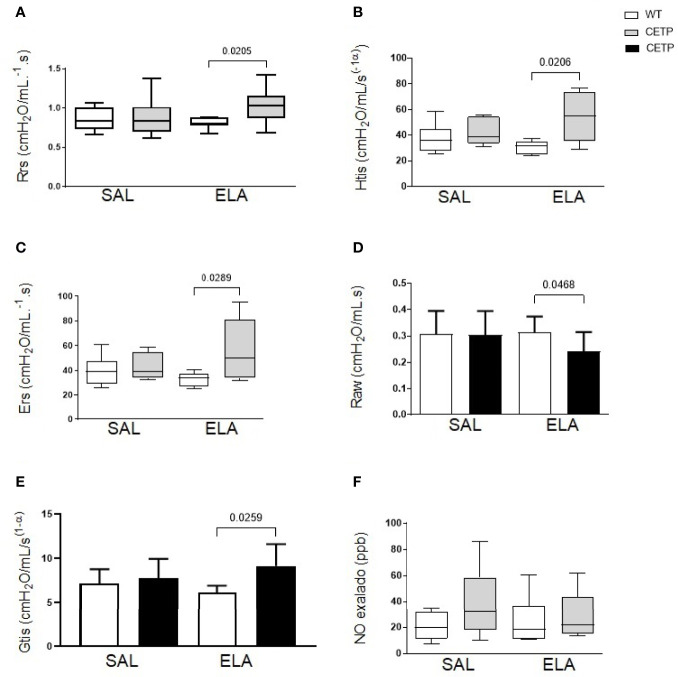
Lung mechanics in WT and huCETP mice after elastase-induced emphysema (ELA), or saline (SAL). **(A)** Respiratory system resistance (Rrs); **(B)** Tissue elastance (Htis); **(C)** Respiratory system elastance (Ers); **(D)** Airway resistance (RAW). **(E)** Lung tissue resistance (Gtis); **(F)** Exhaled nitric oxide. Values expressed as median and percentiles 25 and 75 were analyzed by Kruskal–Wallis test with original FDR method of Benjamini and Hochberg post-test. Values were expressed as mean and standard deviation analyzed by ANOVA with two-stage linear step-up procedure of Benjamini, Krieger, and Yekutieli post-test; (n = 8). P < 0.05.

Considering the importance of macrophages in the development and formation of pulmonary emphysema and its dependence on the microenvironment where they may present an M1 or M2 phenotype, we evaluated the effect of CETP on elastase-induced emphysema (ELA) in CETP Tg and WT mice. After 28 days, we analyzed the lung mechanics, exhaled nitric oxide, total and differential bronchoalveolar lavage (BAL) leukocytes, cytokine production, and mouse lung histology (**Figures 9** **–** **13** and **Table 1**).

**Figure 10:**
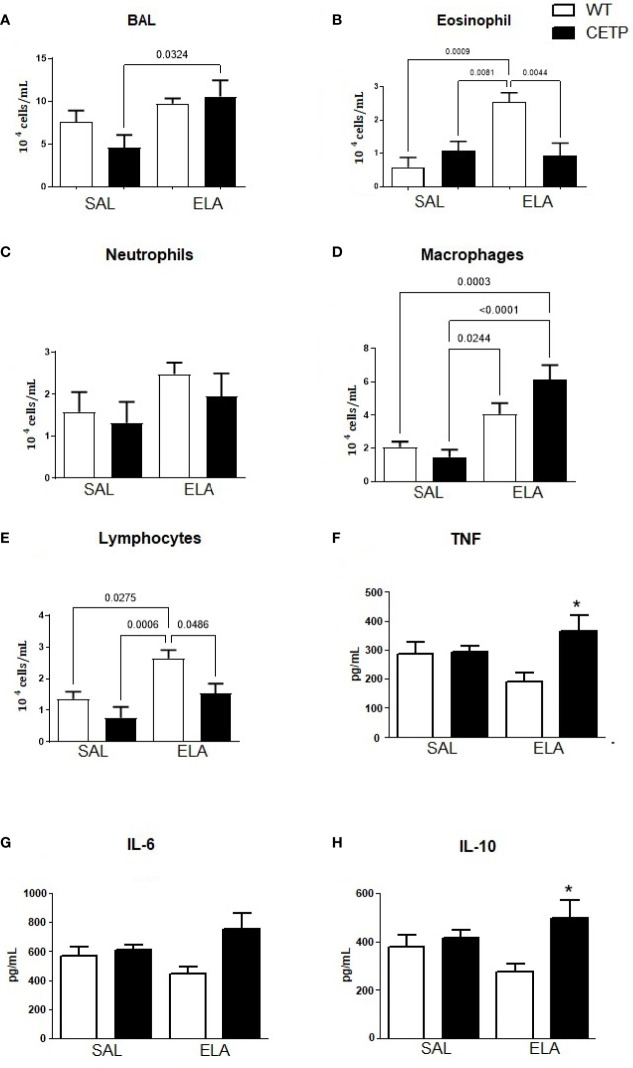
Total and differential leukocyte count in broncho-alveolar lavage (BAL), and cytokine levels in WT and huCETP mice after elastase-induced emphysema (ELA) or saline (SAL). Total cells **(A)**, eosinophils **(B)**, neutrophils **(C)**, macrophages **(D)**, lymphocytes **(E)**, cytokines in BAL: TNF **(F)**, IL-6 **(G)**, IL-10 **(H)**, cytokine plasma **(I)**. Values were expressed as mean and standard deviation. These data were analyzed by ANOVA with two-stage linear step-up procedure of Benjamini, Krieger, and Yekutieli post-test. P < 0.01: *CETP Ela *vs*. all groups, (n = 8).

**Figure 11:**
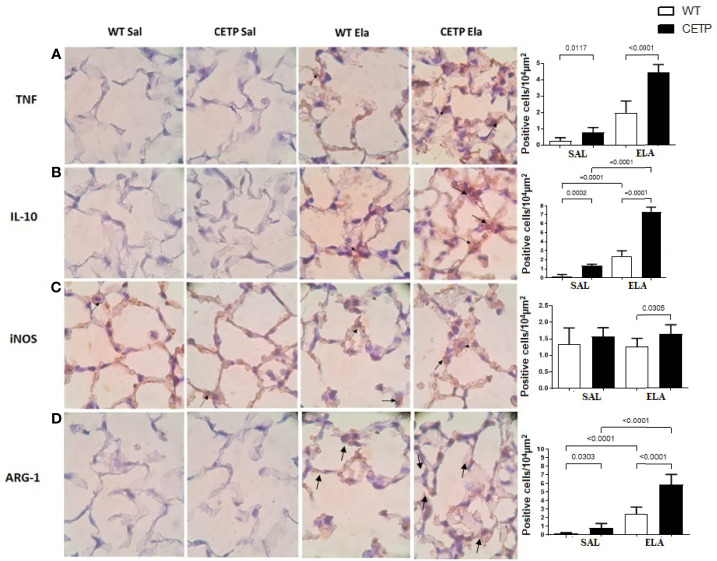
Photomicrograph of stained slides for immunohistochemistry analysis of inflammatory markers from WT and CETP Tg mice and from huCETP mice after elastase-induced emphysema (ELA) or saline (SAL). TNF **(A)**, IL-10 **(B)**, iNOS **(C)** and Arginase 1 **(D)**. Representative images of micrographs at ×1,000 magnification. Values were expressed as mean and standard deviation. These data were analyzed by ANOVA with two-stage linear step-up procedure of Benjamini, Krieger, and Yekutieli post-test. p < 0.01: (n = 8).

**Figure 12:**
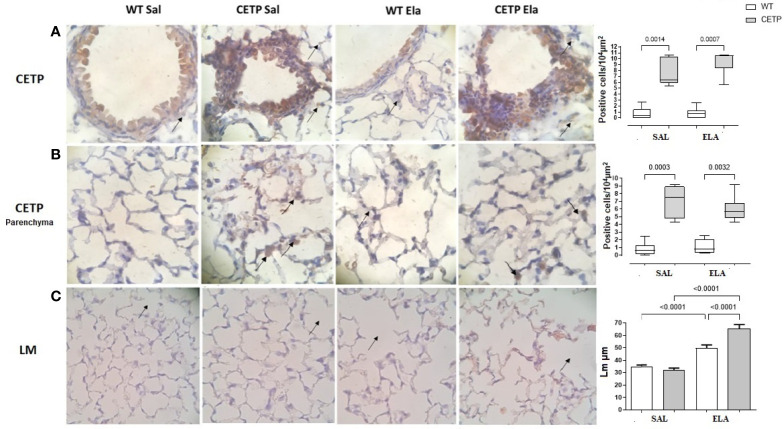
Photomicrograph of stained slides for immunohistochemistry analysis of CETP in airways **(A)**, lung parenchyma **(B)** and mean linear intercept (LM) by dot count of airway spreads **(C)** from WT and huCETP mice after elastase-induced emphysema (ELA) or saline (SAL). Representative images of photomicrographs: CETP magnification ×1,000 and (LM) ×400. Values expressed as median and percentiles 25 and 75 were analyzed by Kruskal–Wallis test with original FDR method of Benjamini and Hochberg post-test. The values were expressed as mean and standard deviation were analyzed by ANOVA with two-stage linear step-up procedure of Benjamini, Krieger, and Yekutieli post-test. p < 0.01, (n = 8).

**Figure 13:**
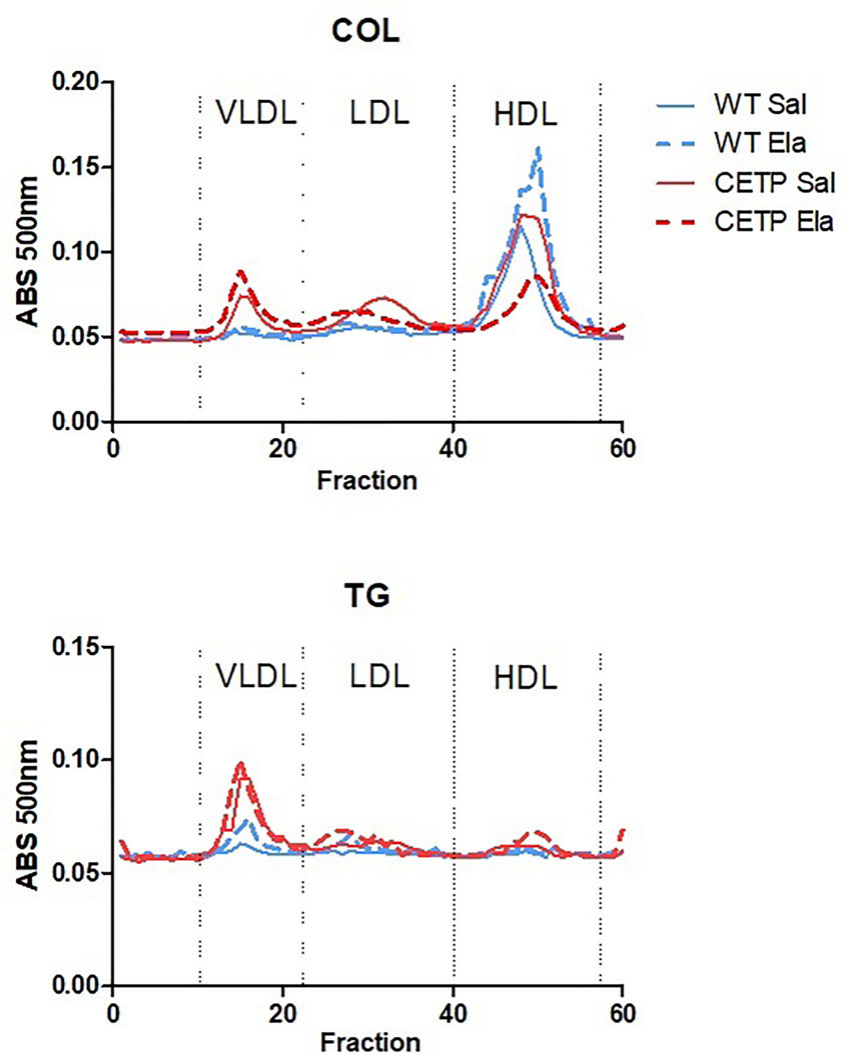
Plasma lipoprotein analysis by high-performance liquid chromatography (FPLC) in WT and huCETP mice after elastase-induced emphysema (ELA) or saline (SAL). Average of two to three pools (n = 4).

**Table 1:** Total cholesterol (C), triglyceride (TG), lipoprotein fractions, cytokines and CETP plasma levels in WT and huCETP mice after elastase-induced emphysema (ELA), or saline (SAL).

	WT SAL	WT ELA	CETP SAL	CETP ELA
CHOLESTEROL TOTAL	73 ± 10	64 ± 8	73 ± 9	65 ± 3
VLDL (mg/dL)	5	4	13	24
%	7	5	17	37
LDL (mg/dL)	13	7	15	12
%	18	11	21	18
HDL (mg/dL)	55	53	45	29
%	75	84	62	45
TRIGLYCERIDE	102 ± 30	123 ± 10	100 ± 20	122 ± 21
VLDL (mg/dL)	51	73	76	80
%	50	59	76	66
LDL (mg/dL)	27	37	10	19
%	26	30	10	15
HDL (mg/dL)	24	13	14	23
%	24	11	14	19
Cytokines (pg/mL)				
IL-6	2,742 ± 175.20	2,561 ± 601.40	2,029 ± 330.50	3,055 ± 213.70*
IL-10	204.1 ± 220.7	189.6 ± 91.3	233.1 ± 52.7	114.5 ± 85.5
CETP				
Concentration (µg/mL)			1.882 ± 0.55	1.907 ± 0.40
Activity (%)			17 ± 4	23 ± 3*

*p < 0.05: CETP ELA vs. CETP SAL; (n = 8).

Elastase administration increased respiratory system resistance (Rrs), tissue elastance (Htis), lung tissue resistance (Gtis), respiratory system elastance (Ers) in CETP mice (**Figures 9A–C, E**), accompanied by a reduction in airway resistance (Raw) compared to the WT-ELA group (**Figure 9D**). There was no difference in exhaled NO analysis (**Figure 9F**).

Bronchoalveolar lavage (BAL) leukocyte differential and total number were measured to analyze the extent of pulmonary inflammation. Exposure to ELA significantly increased macrophage cells in both groups (**Figure 10D**), indicating increased pulmonary inflammation. Total cell levels from CETP-ELA were more abundant than CETP-SAL (**Figure 10A**). Eosinophils and lymphocytes were higher in the WT-ELA group than all experimental groups (**Figures 10B, E**). Increased neutrophil quantity trend was observed in the ELA group (*P* < 0.07) (**Figure 10C**). Significantly increased BAL TNF and IL-10 were observed in CETP-ELA compared to all experimental groups (**Figures 10F, H**). There was no difference in IL-6 (**Figure 10G**).

Lung immunohistochemistry analysis showed increased IL-10, ARG-1, and mean linear intercept (LM) after ELA treatment (**Figures 11B, D** and **12C**) and even higher in CETP-ELA compared to all groups. These results were accompanied by increased TNF, iNOS, and presence of CETP in the lung (**Figures 11A, C** and **12A, B** **)**.

Plasma lipid levels were not modified on elastase infusion. However, induced cholesterol distribution changed in the LP and increased CETP activity (**Figure 13** and **Table 1** **).**

## Discussion

In previous studies, we demonstrated a greater survival rate in human CETP transgenic mice than in WT mice with experimental sepsis (7, 8). CETP along with lipoprotein acceptors, previously recognized key vehicles for the transport of LPS, plays a fundamental role in the initial reversible phase of sepsis and in the regulation of the inflammatory response triggered by LPS in macrophages (5, 7, 8). However, these results diverge from recent studies claiming CETP worsens inflammation and sepsis (9–11). Thus, awareness of the environmental conditions that promote the pro- or anti-inflammatory properties of macrophages and control the plasticity of the phenotype is fundamental for understanding the tissue-specific CETP role (14).

In the present study, we explored the influence of CETP on the differentiation and function of macrophages as well as COPD pathogenesis in a murine model of elastase-induced pulmonary emphysema.

As expected, macrophages derived from huCETP mice expressed CETP (**Figures 1** and **5D**). Polarizing conditions reduced mRNA CETP expression (**Figure 5D**), although the CETP content in the culture medium did not differ between M1 and M2 (**Figure 5E**). Previous study demonstrated reduced expression of CETP in the presence of LPS (27).

After polarization of M0 macrophages with M1 and M2 stimuli, M1 polarized macrophages were confirmed by NOS2, TNF, IL-6, and IL-1β gene expression, and M2 polarized macrophages increased arginase (ARG1), YM1, mannose receptor CD206 (MR), peroxisome proliferator-activated receptor (PPAR) gamma, and IL-10, considered markers of M2 macrophages (*P* < 0.05; M1 *vs*. M2), although it did not differ between WT and CETP groups (**Figure 2**). However, reduced polarization towards M1 was observed when analyzing the expression of surface markers for M1 (CD80) macrophages that produce CETP or WT in the presence of recombinant CETP (**Figures 4A** and **7A, C**) whereas the expression of M2 marker mannose receptor (CD206) was higher than that of WT-M1 (**Figures 4B** and **7B**). Furthermore, the gene expression of mannose receptor (MR: CD206) increased in the presence of recombinant CETP, corroborating these findings (**Figure 6**).

Cytokine profiling revealed increased TNF and L-6 (M1) in both groups as expected (**Figures 5A, B** and **8A, B** **)**. IL-10 has been considered a hallmark of M2 polarization, mainly based on studies in mice (28). However, recent findings demonstrated greater IL-10 expression in M1 than in M2-polarized human monocyte-derived macrophages hMDMs (19, 29). We showed significantly increased IL-10 M1 in the presence of CETP (**Figure 8C**). Considering the diversity of alternatively activated M2 macrophages into subtypes M2a, M2b, M2c, and M2d (22, 30), we speculate that CETP alters the macrophage phenotypic profile (31). This effect can be attributed to toll-like receptor (TLR) activation *via* lipopolysaccharide (LPS) or interferon (IFN) stimulated activation and release of IL-27, with consequent activation of phosphorylated Jak/STAT1 and STAT3 transcription factors, leading to IL-10 production (28). IL-10 gene transcription was greater in M1 and accompanied by increased STAT3 (**Figure 6**).

Anti-inflammatory cytokines increase, initiating an inflammation-controlling process and bacteria elimination. Additionally, anti-inflammatory IL-10 increases to hold back the organ damage excessive inflammation. Consequently, IL-10 often rises following TNF. These results reinforce that IL-10 and TNF are associated in macrophage polarization observed in the CETP group (**Figures 8A, C** and **11A, B**) (32).

M1 and M2 phenotype polarizations represent extremes of activation states. Functional distortion of mononuclear phagocytes occurs *in vivo* under physiological conditions, pregnancy, and in pathologies as allergies, chronic inflammation, tissue repair, infection, and cancer (33). M1 to M2 repolarization exerted by distinct M2 stimuli through transcriptome-based path analysis provides a new approach to human macrophage phenotypes in clinically relevant disease states, such as cystic fibrosis and asthma (34).

According to the hypothesis that CETP plays an important role in immunomodulation and favors a lower M1 macrophage profile, a significant increase of IL-10 in CETP compared to WT furthers our understanding of the dual role of CETP in different conditions, namely: 1) its anti-inflammatory profile conferring longer survival in endotoxemia in CETP mice (7, 8), although this has been contested (9), and (2) worsening inflammation and tissue damage in the experimental elastase-induced pulmonary emphysema model which resulted in the widening of alveolar air spaces (Lm) in CETP animals compared to WT eliciting pulmonary emphysema worsening (**Figure 12C**).

We demonstrated reduced LPS uptake in the presence of both endogenous and exogenous CETP compared to WT (7, 8). Increased activation of NF-k beta with macrophage TNF-alpha production was observed in the absence of CETP (8). Another study showed that cholesterol accumulation in macrophages increased the inflammatory response mediated by toll-like 4 receptors (TLR4s), exacerbating LPS-mediated secretion of pro-inflammatory cytokines (35). This indicates that cellular cholesterol concentration has a direct influence on immunoregulation. Thus, the recruitment of TLR4 to lipid-rafts is due to increased cholesterol in these membrane microdomains. We speculate that CETP reduces cellular cholesterol microdomains impairing TLR4 recruitment. In this regard, increased ABCA1 transcription was observed in CETP-M2 compared to WT-M2 (**Figure 3**). ABCA1 promoted cholesterol efflux from macrophage and increased IL-10. Moreover, it reduces the secretion of pro-inflammatory cytokines, activating PKA and contributing to the M2 type anti-inflammatory response (36, 37).

Several other gene expressions that connect lipid metabolism and inflammation, like LXR, PPAR gamma, and SREBP, did not differ between the experimental groups. However, SRB1 (SCARB) increased after M2 polarization (**Figure 3**) SRB1 is a well-characterized lipoprotein receptor that binds native high- and low-density lipoproteins (HDL and LDL), respectively (38). SRB1 also plays an important role in the recognition of other endogenous and exogenous ligands, inducing an anti-inflammatory or pro-inflammatory response depending on the nature of the ligand and the cell/tissue type (39). Our data are in accordance with a study that shows increased SR-B1 mRNA levels in THP-1 cell after M2 polarization compared to M1.

It is known that the hepatic expression of CETP is regulated by LXR (40). LXR agonists attenuate LPS-induced release of TNF alpha and prostaglandin E2 from isolated Kupffer cells (41), suggesting that LXRs keep the pro-inflammatory activation status of Kupffer cells low, which coincides with high CETP expression. Reduced mRNA CETP expression was observed in the generation of M1 and M2 macrophages under polarizing conditions (**Figure 5D**).

In the induction of pulmonary emphysema by elastase instillation (ELA), worsening of the lung mechanical parameters in the CETP ELA group (**Figures 9A–C, E**) was accompanied by a reduction in airway resistance (Raw) compared to the WT-ELA group (**Figure 9E**) These mechanical changes are consistent with the increase in the mean linear intercept, which is reflected in the widening of the air spaces owing to the destruction of the lung parenchyma in the CETP ELA (**Figure 12C**).

Cytokines in BAL revealed increased IL-10 compared to WT ELA, corroborating the CETP ELA lung immunohistochemistry data (**Figures 10H** and **11B**).

Eosinophils and lymphocytes were higher in WT ELA than in all experimental groups (**Figures 10B, E**). The role of eosinophils in the pathogenesis of COPD is still controversial, and many cases occur with tissue eosinophilia without a simultaneous increase in blood eosinophils (42). Neutrophils showed a trend to increase in ELA groups (P < 0.07) (**Figure 10C**). Increase neutrophil levels are characteristic of acute exacerbations of COPD (43). The exhaled NO (**Figure 9F**) was not different between the experimental groups, probably because of large individual variation. However, iNOS labeling in lung tissue was greater in CETP-ELA **(** **Figure 11C** **).**

Elevation of plasma IL-6 (pg/ml) (P < 0.05) and low IL-10 (P < 0.07) (**Table 1**) occurred in the CETP-ELA group, possibly indicating systemic inflammatory response after elastase. Patients with severe COPD showed a higher IL-6/IL-10 ratio (44).

In contrast to plasma data, IL-10 was elevated in the CETP-ELA in the lungs and was accompanied by a higher expression of Arg-1, markers of M2-type macrophages (**Figures 11B, D** **)**. Increased Arg-1 suggests an aggravation of catabolic activity but may also have implications for lung fibrosis and airway resistance (45). These data corroborate those of *in vitro* macrophage polarization towards the M2 type in the presence of CETP and are potentially important for understanding the pathophysiology of the respiratory tract response in the presence of CETP and the etiology of COPD.

Plasma lipid levels were not modified on elastase infusion (**Table 1**). However, elastase-induced cholesterol distribution changes in the LP of CETP and WT mice that were distinct according to genotype (**Figure 13** and **Table 1**). As expected, in huCETP mice, HDL-C decreased, and VLDL-C slightly increased. However, after elastase administration, substantial HDL-C reduction occurred, which is explained by increased CETP activity; these results were independent of plasma CETP levels (**Table 1**). These results indicate that CETP expression in the elastase group led to unfavorable modifications, with increased apoB-LP and reduced HDL contributing to the worsening inflammation in CETP ELA group. In humans, there was no significant difference in the concentrations of LDL, apolipoprotein B, or HDL between groups with mild, moderate, and severe COPD. Triglyceride concentrations were reduced, and apoA1 increased in the most severe cases (46).

Our results are in agreement with those of another investigation showing alveolar macrophages with a dominant M2 phenotype in patients with COPD (42). Another study found increased deposition of M2 alveolar macrophages in the mouse model of COPD and increased expression of the TGF beta/Smad pathway in M2 macrophages, both *in vitro* and *in vivo*, indicating that M2 macrophages contribute to COPD by altering its phenotypic profile (26).

Although polarization to M2 cannot be attributed to CETP alone, its presence exacerbated the effects of elastase. These findings suggest that the dominant accumulation of M2 in the presence of CETP in the lung should be explored in COPD (47). Interestingly, the accumulation of M2 macrophages was observed in adipose tissue and liver, in tissues with high expression of CETP and the lung, as well as in the blood of patients who suffered severe trauma, such as burns or sepsis. However, the induction of M2 polarization in these patients has not yet been identified (48). In this sense, it was recently suggested that CETP inhibitors be redirected to the treatment of sepsis (49).

Despite the limitations of animal model observations for human physiology, our results are novel, indicating that CETP is associated with the inflammatory response and, especially, with the role of macrophages in COPD.

## Data Availability Statement

The raw data supporting the conclusions of this article will be made available by the authors, without undue reservation.

## Ethics Statement

The animal study was reviewed and approved by Ethical Committee of the University of São Paulo Medical School for the use of animals (CEUA 949/2018).

## Author Contributions

KS performed all the experiments and data analysis and helped to write the article. RR helped with animal and immunohistochemistry experiment. CS helped with flow cytometry analyses and statistics. OA helped with flow cytometry analyses. TR helped with data interpretation and molecular biology. FD helped with animal experiments and CETP genotyping mice. VN helped with the experiments to analyse the lipid profile and chromatography. IT helped with animal experimental design. FG helped in the experimental design. NC helped in the study design and data interpretation EQ helped in data interpretation and in writing the article. PC supervised the experimental design, data interpretation, and writing the article, and was awarded the research funding. All authors contributed to the article and approved the submitted version.

## Funding 

The authors would like to thank the financial support from Fundação de Amparo à Pesquisa do Estado de São Paulo, FAPESP (grants # 2017/22940-6 to PC).

## Conflict of Interest

The authors declare that the research was conducted in the absence of any commercial or financial relationships that could be construed as a potential conflict of interest.
