# Supplementary material for: Cholesterol-Ester Transfer Protein Alters M1 and M2 Macrophage Polarization and Worsens Experimental Elastase-Induced Pulmonary Emphysema
Source: Front Immunol. 2021 Jul 21;12:684076. doi: 10.3389/fimmu.2021.684076 (PMC8334866; doi:10.3389/fimmu.2021.684076)
Supplement: Supplementary file 1 [file DataSheet_1.pdf]

Supplementary Material and Methods

Mice Genotyping Analysis

Mice genotyping was performed through the collection of distal tail tissue (tail biopsy), followed by genomic DNA extraction using the HotSHOT protocol (Truett, G.E. et al. Biotechniques, 2000. 29: p.52-5). The presence of the human CETP gene was confirmed by conventional polymerase chain reaction (PCR), according to the Jackson Laboratory-modified protocol for the B6.CBA-Tg (CETP) 5203Tall / J line, number 3904 (2009), using GoTaq® DNA Polymerase for amplification. Sequences of primers used to amplify the CETP gene are as follows: (Forward: GAATGTCTCAGAGGACCTCCC; Reverse: CTTGAACTCGTCTCCCATCAG), control (Forward: CTAGGCCACAGAATTGAAAGATCT; Reverse: GTAGGTGGAAATTCTAGCATCATCC) (1, 2).

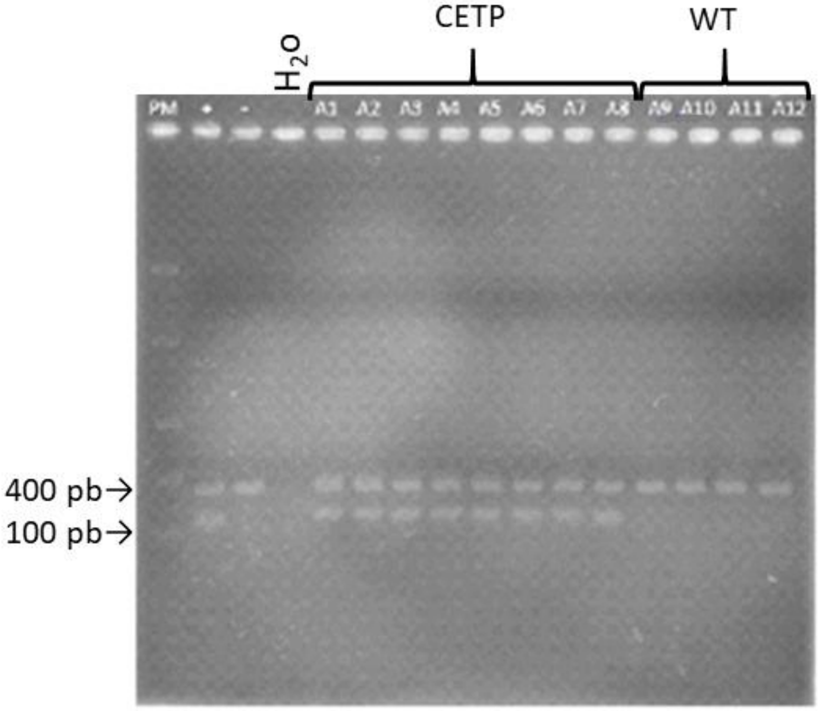

Figure 1. Screening of human CETP transgenic and non-transgenic wild type (WT) littermates. Gel electrophoresis (1.5% agarose) showing PCR profiles of amplified DNA from mice. The positive human CETP gene (160 bp) samples are present in A1 to A8 and the negative control gene (320 bp) samples in A9 to A12.

CETP Activity Assay

Plasma CETP activity was measured by an exogenous method that mirrors the plasma CETP concentration, as previously described (2). Briefly, a mixture of lipoproteins (LP) from human donors, very low density lipoprotein (VLDL), and low density lipoprotein (LDL) (200 mg/dL cholesterol) was incubated with high density lipoprotein (HDL) previously labelled with [4-14C]-CE (40 mg/dL cholesterol) in the presence of mouse plasma (10 μL) as the source of CETP plus Tris buffer (40 μL); final volume 300 μL. Blanks were prepared with Tris/saline/EDTA buffer (10/140/1 mM, pH 7.4), and negative controls with WT mouse plasma. Incubations were carried out at 37°C for 2 h. Then, the apoB-LP (VLDL and LDL) was precipitated with 1.6% dextran sulfate/1 M $MgCl_2$ solution (1:1), and radioactivity was measured in the remaining supernatant in a scintillation solution (Ultima Gold, Eastman Kodak Co., Rochester, NY) in the LS6000 Beckman Beta Counter (Beckman Instruments, Palo Alto, Calif). The percentage of [4-14C]-CE transferred from [14C]-CE-HDL to VLDL + LDL was calculated as [1 - (sample radioactivity/control radioactivity) × 100] (3).

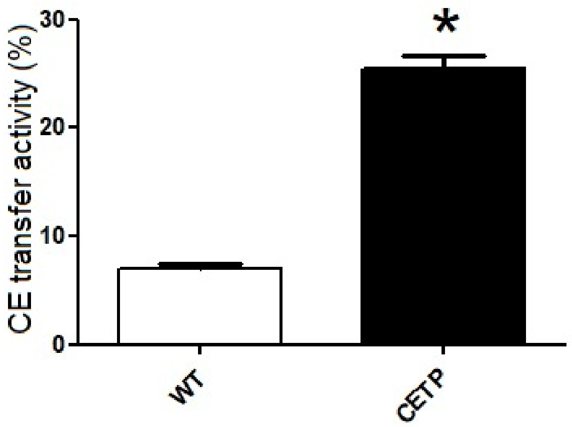

Figure 2. Plasma CETP activity in CETP Tg and WT mice. Animals were characterized according to their respective phenotypes: plasma CETP activity (>20% CETP Tg mice and < 10% in WT). Statistical analysis was performed using: Mann Whitney test; WT vs. CETP. *p< 0.0001. WT (n=28), CETP (n=33).

Lipids and Plasma Lipoprotein

Plasma lipoproteins from pooled mouse plasma were separated by gel filtration using two Superose 6 HR 10/30 columns connected in series (Amersham-Pharmacia Biotech., Uppsala, Sweden). Lipoproteins were eluted at a constant flow rate of 0.5 mL/min with Tris buffer (10 mM Tris, 150 mM NaCl, 1 mM EDTA and 0.03% NaN_3, pH 7.0). The fractions (0.2 ml) were collected in 96-well plates using the fraction collector. Total cholesterol (TC) and triacylglycerol (TG) content was determined by enzymatic-colorimetric method using Labtest kits (Labtest Diagnóstica - Minas Gerais - Brazil) according to the manufacturer's instructions. The peaks corresponding to lipoproteins VLDL, LDL, and HDL were identified by measuring the absorbance of the TC (4).

.

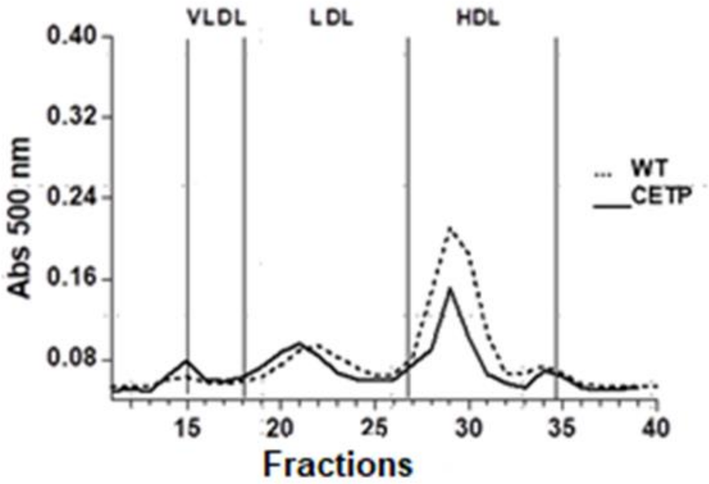

Figure 3. Plasma lipoprotein profile of CETP and WT mice. Peaks corresponding to lipoproteins VLDL, LDL, and HDL, isolated by FPLC were identified by measuring absorbance of the total cholesterol (TC) at 500 nm using the enzymatic-colorimetric method. (n=8).

As expected, CETP mice showed a decrease in HDL-C and a slight increase in VLDL-C.

GENE	PROBE CODE (TAQMAN)
ABCA1	Mm00442646_m1
ARG1	Mm00475988_m1
β –actina	Mm02619580_g1
Casp3	Mm01195085_m1
Cd36	Mm00432403_m1
CETP	Hs00163942_m1
Il10	Mm01288386_m1
Il18	Mm00434225_m1
Il1b	Mm00434228_m1
Il6	Mm00446190_m1
Nos2 (iNOS)	Mm01309902_m1
Nr1h2 (LXR)	Mm00437265_g1
Pparg (PPARγ)	Mm00440940_m1
Scarb1 (SRB1)	Mm00450234_m1
Srebf1 (SREBP)	Mm00550338_m1
TNF	Mm00443258_m1
**GENE**	**PRIMER SEQUENCE (SYBR)**
Hprt F	AGCAGGTCAGCAAAGAACT
Hprt R	CCTCATGGACTGATTATGGACA
MR F	CAAGTTGCCGTCTGAACTGA
MR R	TATCTCTGTCATCCCTGTCTCT
Nnt-COM	GTAGGGCCAACTGTTTCTGCATGA
Nnt-MUT	GTGGAATTCCGCTGAGAGAACTCTT
Nnt-WT	GGGCATAGGAAGCAAATACCAAGTTG
Stat3 F	GGAAATAACGGTGAAGGTGCT
Stat3 R	CATGTCAAACGTGAGCGACT
Ym1 F	ACTGGTATAGTAGCACATCAGC
Ym1 R	AGAAGCAATCCTGAAGACACC

Table 1. Probes and primers used to determine gene expression by RT-PCR.

MARKER	PRIMARY ANTIBODY	DILUTION	SPECIFICATION
TNF	Monoclonal produced in mouse	1:100	SC-52746, Sta. Cruz Biotechnology, CA, USA
IL-10	Monoclonal produced in rat	1:50	SC-73309, Sta. Cruz Biotechnology, CA, USA
iNOS	Polyclonal produced in rabbit	1:500	12613507, Labvision, Neon markers, CA, USA
Arg-1	Monoclonal produced in mouse	1:50	610708, BD Biosciences, United Kingdom.
CETP	Polyclonal produced in rabbit	1:200	ab51771, Abcam, USA.

Table 2. Antibodies used to determine inflammatory and anti-inflammatory markers by immunohistochemistry.

1. M. Hoekstra, D. Ye, R. B. Hildebrand, Y. Zhao, B. Lammers, M. Stitzinger, J. Kuiper, T. J. C. Van Berkel and M. Van Eck: Scavenger receptor class B type I-mediated uptake of serum cholesterol is essential for optimal adrenal glucocorticoid production. *Journal of Lipid Research*, 50(6), 1039-1046 (2009) doi:10.1194/jlr.M800410-JLR200

2 R. McPherson, C. J. Mann, A. R. Tall, M. Hogue, L. Martin, R. W. Milne and Y. L. Marcel: PLASMA-CONCENTRATIONS OF CHOLESTERYL ESTER TRANSFER PROTEIN IN HYPERLIPOPROTEINEMIA - RELATION TO CHOLESTERYL ESTER TRANSFER PROTEIN-ACTIVITY AND OTHER LIPOPROTEIN VARIABLES. *Arteriosclerosis and Thrombosis*, 11(4), 797-804 (1991)

3 P. M. Cazita, J. A. Berti, C. Aoki, M. Gidlund, L. M. Harada, V. S. Nunes, E. C. R. Quintao and H. C. F. Oliveira: Cholesteryl ester transfer protein expression attenuates atherosclerosis in ovariectomized mice. *Journal of Lipid Research*, 44(1), 33-40 (2003) doi:10.1194/jlr.M100440-JLR200

4. R. M. Machado, J. T. Stetano, C. Oliveira, E. S. Mello, F. D. Ferreira, V. S. Nunes, V. M. R. de Lima, E. C. R. Quintao, S. Catanozi, E. R. Nakandakare and A. M. P. Lottenberg: Intake of trans Fatty Acids Causes Nonalcoholic Steatohepatitis and Reduces Adipose Tissue Fat Content. *Journal of Nutrition*, 140(6), 1127-1132 (2010) doi:10.3945/jn.109.117937
